# Micropapular Cutaneous Sarcoidosis Confined to the Face: An Uncommon Clinical Variant

**DOI:** 10.7759/cureus.85135

**Published:** 2025-05-31

**Authors:** Sonia Selvaraj, Ambigai SSK, Sudha Rangarajan, Leena Dennis Joseph, Adikrishnan Swaminathan

**Affiliations:** 1 Dermatology Venereology Leprosy, Sri Ramachandra Institute of Higher Education and Research, Chennai, IND; 2 General Pathology, Sri Ramachandra Institute of Higher Education and Research, Chennai, IND

**Keywords:** cutaneous sarcoidosis, facial papules, granulomatous dermatoses, micropapular sarcoidosis, noncaseating granuloma

## Abstract

Micropapular sarcoidosis represents an uncommon variant of cutaneous sarcoidosis, typically presenting with a sudden onset, often resolving without residual scarring, and generally favorable prognosis. Its resemblance to other chronic papular dermatoses makes diagnosis challenging, especially in cases with subtle or localized cutaneous manifestations. We report a case of a 36-year-old female who presented with gradually progressive, asymptomatic skin-colored, and erythematous papules localized to the periorbital and perioral regions over six months. Systemic symptoms were absent, and routine blood investigations revealed anemia and elevated erythrocyte sedimentation rate (ESR). Skin biopsy revealed noncaseating epithelioid granulomas with lymphocytes suggestive of cutaneous sarcoidosis. The patient responded well to a combination of oral corticosteroids, hydroxychloroquine, and topical corticosteroids, with near-complete resolution of lesions and residual post-inflammatory hyperpigmentation over a period of three months. This case emphasizes the importance of considering micropapular sarcoidosis in the differential diagnosis of chronic facial papular eruptions and highlights the effectiveness of early appropriate treatment.

## Introduction

Sarcoidosis is a chronic multisystem granulomatous disease of unknown etiology that can affect any organ, with the lungs and skin being the most commonly involved sites. Cutaneous involvement occurs in approximately 25-30% of patients with systemic sarcoidosis, although in some cases, it may present as an isolated skin condition [1]. Sarcoidosis displays a protean spectrum of skin presentations, some of which include papules, plaques, nodules, lupus pernio, scar infiltration, ichthyosis, and erythroderma [2]. Micropapular sarcoidosis is a rare variant, typically manifesting as tiny grouped papules [3]. It may mimic other dermatological conditions such as syringoma, comedonal acne, papular granuloma annulare, and lichen nitidus, thereby posing a diagnostic challenge. Histologically, the hallmark is the presence of noncaseating epithelioid granulomas [4]. The clinical course is usually benign, and treatment with corticosteroids and antimalarials is often effective [3]. Early recognition of this rare presentation is crucial for prompt treatment and to avoid unnecessary investigations and therapeutic delays.

## Case presentation

**Figure 1:**
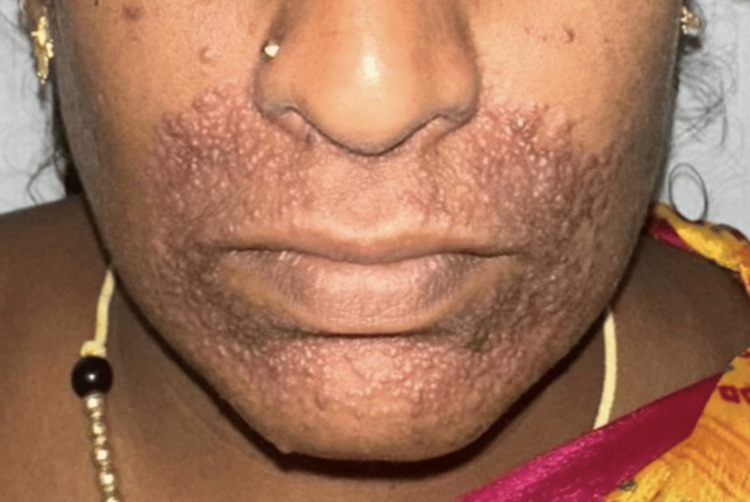
Multiple firm, skin-colored and erythematous, shiny, non-scaly 1 mm papules over the perioral area, with some confluence forming 2-3 mm papules.

A 36-year-old female presented to the dermatology outpatient department with asymptomatic progressive red-colored and skin-colored lesions over her eyelids that progressed to involve the cheeks and perioral region over the past six months. There was no history of systemic symptoms such as fever, weight loss, cough, night sweats, or fatigue. There were no similar lesions elsewhere on the body, and there was no personal or family history of tuberculosis or systemic illness. On cutaneous examination, multiple firm, skin-colored and erythematous, shiny, non-scaly, non-necrotic 1 mm papules were noted over the eyelids and perioral area, with some confluence forming 2-3 mm papules, as shown in Figure 1. Based on the clinical morphology and distribution, differential diagnoses considered were syringoma, lupus miliaris disseminatus faciei, xanthoma, micropapular sarcoidosis, and papular syphilide.

**Figure 2:**
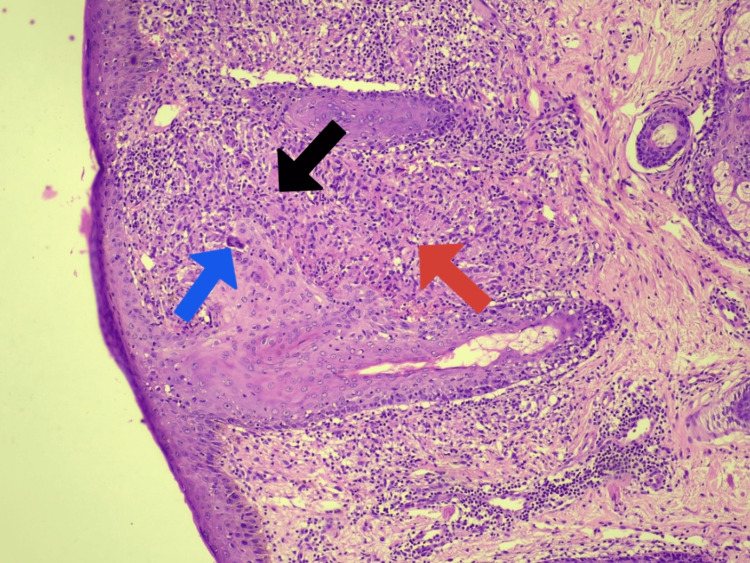
Histopathology image of the biopsy from papule in the perioral region. The epidermis shows focal atrophy and flattening of the rete ridges. The upper dermis exhibits focal epithelioid granulomas (red arrow) admixed with multinucleated giant cells (blue arrow) and lymphocytes (black arrow).

Routine hematological investigations revealed decreased hemoglobin and elevated ESR; normal liver function tests, renal function tests, angiotensin-converting enzyme (ACE) levels, calcium levels, and vitamin D levels; a non-reactive venereal disease research laboratory (VDRL) test; negative treponema pallidum hemagglutination assay (TPHA); and a negative Mantoux test (0 mm induration), as shown in Table 1. Chest X-ray and abdominal ultrasonography were normal. A punch biopsy taken from a papule showed focal atrophy of the epidermis with flattening of the rete ridges. The upper dermis exhibited non-caseating epithelioid granulomas admixed with multinucleated giant cells and lymphocytes, as shown in Figure 2. Periodic acid-Schiff (PAS) and acid-fast bacilli (AFB) stains were negative.

**Table 1:** Investigation values in the patient.

Investigations	Values	Normal/range values
Hemoglobin	11.7 g/dL	12-15 g/dL
Erythrocyte sedimentation rate	16 mm/hr	4-12 mm/hr
Serum calcium	9.8 mg/mL	8.8-10.6 mg/dL
Serum vitamin D	32.76 ng/dL	30-70 ng/dL
Serum angiotensin-converting enzyme	49 U/L	13.3-63.9 U/L

The absence of comma-shaped or tadpole-shaped eccrine ducts embedded in dense fibrous stroke in histopathological examination (HPE) ruled out syringoma, absence of caseating perifollicular epithelioid granuloma ruled out lupus miliaris disseminatus faciei, absence of foamy histiocytes ruled out xanthoma, and negative VDRL ruled out papular syphilide. Based on the characteristic clinical and histopathological features, a diagnosis of micropapular sarcoidosis was made.

**Figure 3:**
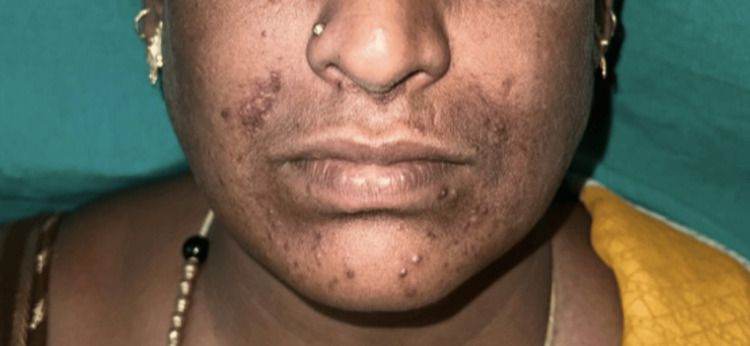
Post-treatment photograph taken after three months shows resolving perioral lesions with post-inflammatory hyperpigmentation.

The patient was started on oral prednisolone 20 mg per day, hydroxychloroquine 200 mg once daily, and topical mometasone furoate 0.1% for local application. Oral steroids were gradually tapered and stopped by the end of two months. Over a period of two months, there was no recurrence, and significant improvement in the lesions was noted. The patient continues to be maintained on hydroxychloroquine 200 mg once daily. At the end of three months, 80% of the lesions had resolved, leaving post-inflammatory hyperpigmentation as shown in Figure 3. No systemic involvement has been noted to date.

## Discussion

Micropapular sarcoidosis is an uncommon morphological variant of cutaneous sarcoidosis, typically presenting as tiny uniformly grouped papules, with reported cases primarily involving the face and trunk. It is more commonly observed in women around the age of 40 and has been reported across all races, although African American women have the highest incidence in the United States [5,6]. The pathogenesis of sarcoidosis involves an exaggerated immune response to an unidentified antigen, resulting in the formation of noncaseating granulomas [4]. Our patient, a 36-year-old female, presented with asymptomatic red and skin-colored papules distributed primarily on the face, with no systemic complaints, a presentation consistent with other reported cases of micropapular sarcoidosis [7,8].

The absence of systemic features in our case aligns with prior reports indicating that micropapular sarcoidosis can occur as an isolated cutaneous disease. Most published cases have shown no evidence of pulmonary or systemic organ involvement, reinforcing the notion that micropapular sarcoidosis may follow a benign course with an excellent prognosis [9]. Although ocular involvement is noted to be relatively frequent in this subtype, our patient did not demonstrate any ocular findings [8].

Histologically, this variant displays classic features of sarcoidosis, including non-caseating epithelioid granulomas localized to the upper dermis. In our case, the biopsy revealed focal epidermal atrophy with well-formed non-caseating epithelioid granulomas, multinucleated giant cells, and lymphocytes, consistent with a sarcoidal granulomatous reaction pattern [4].

Micropapular sarcoidosis can be challenging to diagnose clinically due to its resemblance to other dermatoses such as lichen nitidus, syringoma, xanthoma, lupus miliaris disseminatus faciei, papular granuloma annulare, and papular syphilide, which were systematically ruled out through clinical, histological, and serological assessment.

Therapeutically, the patient showed an excellent response to systemic corticosteroids and hydroxychloroquine, with 80% resolution of lesions and residual post-inflammatory hyperpigmentation. This outcome is consistent with previous reports where corticosteroids and antimalarials were shown to be effective for isolated cutaneous sarcoidosis without systemic involvement [3]. Other treatment options for refractory cases mentioned in the literature include methotrexate, tetracyclines, dapsone, colchicine, intralesional steroids, and biological agents such as infliximab or adalimumab [10].

## Conclusions

Micropapular sarcoidosis is a rare but important consideration in the differential diagnosis of chronic facial papular eruptions. Its resemblance to other benign dermatoses often results in delayed recognition and treatment. Early diagnosis requires a high degree of clinical suspicion and confirmation through histopathology. Our case highlights that timely identification can lead to excellent therapeutic outcomes and prevent disease progression. Dermoscopy and biopsy serve as valuable diagnostic aids. Initiating appropriate therapy early improves cosmetic and functional outcomes. Greater awareness among clinicians is essential to minimize morbidity and optimize patient care.
